# Tspan5 is an independent favourable prognostic factor and suppresses tumour growth in gastric cancer

**DOI:** 10.18632/oncotarget.9514

**Published:** 2016-05-20

**Authors:** Peirong He, Suihai Wang, Xuefeng Zhang, Yanjun Gao, Wenbo Niu, Ningning Dong, Xiangyi Shi, Yan Geng, Qiang Ma, Ming Li, Bo Jiang, Ji-Liang Li

**Affiliations:** ^1^ School of Biotechnology, Southern Medical University, Guangzhou 510515, China; ^2^ Guangdong Provincial Key Laboratory of Gastroenterology, Department of Gastroenterology, Nanfang Hospital, Southern Medical University, Guangzhou 510515, China; ^3^ Institute of Translational and Stratified Medicine, Plymouth University Peninsula Schools of Medicine and Dentistry, Plymouth PL6 8BU, U.K

**Keywords:** gastric cancer, tetraspanin, tumour suppressor, cell cycle, biomarker

## Abstract

Tetraspanins are believed to interact with specific partner proteins forming tetraspanin-enriched microdomains and regulate some aspects of partner protein functions. However, the role of Tspan5 during pathological processes, particularly in cancer biology, remains unknown. Here we report that Tspan5 is significantly downregulated in gastric cancer (GC) and closely associated with clinicopathological features including tumour size and TNM stage. The expression of Tspan5 is inversely correlated with patient overall survival and is an independent prognostic factor in GC. Upregulation of Tspan5 in tumour cells results in inhibition of cell proliferation and colony formation *in vitro* and suppression of xenograft growth of GC by reducing tumour cell proliferation *in vivo.* Thus, Tspan5 functions as a tumour suppressor in stomach to control the tumour growth. Mechanistically, Tspan5 inhibits the cell cycle transition from G1-S phase by increasing the expression of p27 and p15 and decreasing the expression of cyclin D1, CDK4, pRB and E2F1. The correlation of Tspan5 expression with the expression of p27, p15, cyclin D1, CDK4, pRB and E2F1 *in vivo* are also revealed in xenografted tumours. Reconstitution of either cyclin D1 or CDK4 in Tspan5-overexpressing GC cells rescues the inhibitory phenotype produced by Tspan5, suggesting that cyclin D1/CDK4 play a dominant role in mediating the suppression of tumour growth by Tspan5 in GC. Our results suggest that Tspan5 may serve as a prognostic biomarker for predicting outcome of GC patients and provide new insights into the pathogenesis of GC and rational for the development of clinical intervention strategies against GC.

## INTRODUCTION

Gastric adenocarcinoma (gastric cancer, GC) is the fifth most common malignant disease and third leading cause of cancer-related mortality worldwide [1]. However, in China GC is currently the second most common cancer and second leading cause of cancer death [2]. A total of 679,100 new stomach cancer cases and 498,000 deaths are estimated in 2015, accounting for 15.8% of the total estimated cancer cases and 17.7% of total estimated deaths in China [2]. Although both diagnosis and treatment have been improved [3, 4], overall 5-year survival only ranges from 5-20% with the best median overall survival of 13.8 months so far [5]. The tumour-node-metastasis (TNM) classification is the most widely used staging system for prognosis of GC patients, but it is difficult to obtain precise prognostic information [6]. Therefore, identification of new targets will help us to understand the molecular mechanisms of pathogenesis and to develop novel targeted therapies or biomarkers for evaluating the therapeutic efficacy or predicting the outcome of GC patients [7].

Tetraspanins are evolutionarily conserved small proteins of 204-355 amino acids (20–50kDa) that are characterized by four conserved transmembrane (TM) domains, short cytoplasmic amino- and carboxyl-terminal tails, a short intracellular loop, a small extracellular loop, and a large extracellular loop [8]. Tetraspanins typically organize laterally with specific partner proteins to form tetraspanin-enriched microdomains (TEMs) via tetraspanin-tetraspanin interactions [9, 10]. Within TEMs, tetraspanins are believed to coordinate some aspects of partner protein functions, particularly those of receptor tyrosine kinases (eg EGFR and c-Met), regulating cell adhesion, migration, invasion, signaling, cell-cell fusion, infection by cancer-causing viruses, morphology and survival during physiological and pathological processes [8, 11, 12]. Total 33 members in the tetraspanin superfamily have been identified in humans, but only a limited number of members have so far been studied in mammalian cells. Some members such as Tspan24 (CD151), Tspan28 (CD81), Tspan29 (CD9) and Tspan30 (CD63) have a wide cell and tissue distribution while others such as Tspan20 (UP1b), Tspan21 (UP1a), Tspan22 (RDS), Tspan23 (ROM1), Tspan25 (CD53) and Tspan26 (CD37) show a more restricted pattern of expression [11, 13]. It has been reported that many tetraspanins such as Tspan1 (NET-1), Tspan8 (CO-029), Tspan13 (NET-6), Tspan24 (CD151), Tspan27 (CD82), Tspan28 (CD81), Tspan29 (CD9) and Tspan30 (CD63) were deregulated in various types of human cancers, implying that these tetraspanins may be involved in tumourigenesis and/or tumour progression [11–17]. In contrast, little has been known about Tspan5 (NET-4, TMS4SF9). Tspan5 was reported to highly express in brain cortical structures including the hippocampus, amygdala and in Purkinje cells, and parallel neuronal maturation in cerebellum of mice [18, 19]. Such expression pattern suggests that Tspan5 might be involved in both developmental and functional maturation of the brain [20]. It was reported that osteoclastogenic RANKL signaling could increase Tspan5 expression in osteoclast precursor cells [21] and knockdown of Tspan5 expression inhibited osteoclastogenesis [22], suggesting the role of Tspan5 in osteoclast formation and osteoclast differentiation. However, whether Tspan5 has a role in tumourigenesis is unknown.

We are interested in the role of Tspan5 in cancer biology and clinical significance. In this study, we report that Tspan5 was downregulated in GC and closely associated with clinicopathological features. The expression of Tspan5 was inversely correlated with patient overall survival and was an independent prognostic factor in GC. Upregulation of Tspan5 expression demonstrated that Tspan5 functions as a tumour suppressor to inhibit cell proliferation *in vitro* and xenograft growth *in vivo*. The underlying molecular mechanisms were unveiled to control the cell cycle transition from G1-S phase by regulating the activity in the molecular pathway of p27/cyclin D1/CDK4/pRB/E2F1 in GC.

## RESULTS

### Downregulation and association of Tspan5 with clinicopathological feature of GC

**Figure 1:**
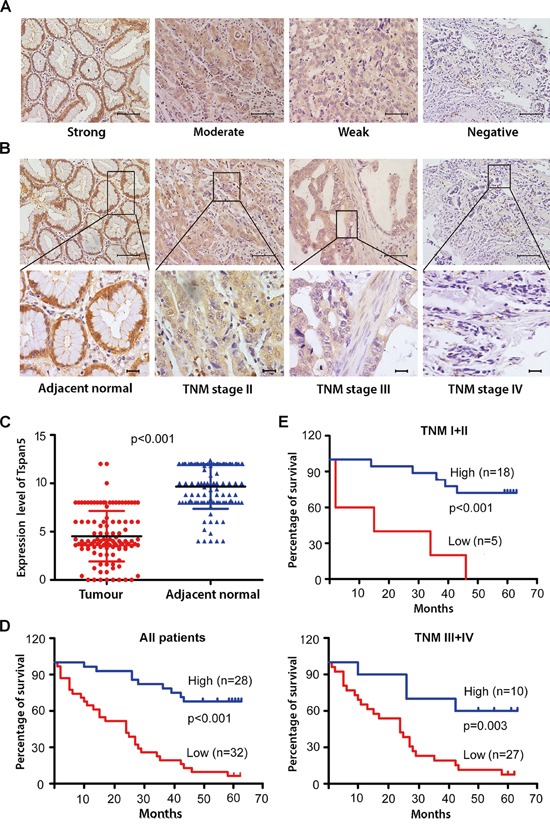
Tspan5 was downregulated in GC and correlated with clinicopathological features and patient overall survival **A.** IHC analysis of Tspan5 expression in 114 pairs of tumour tissues and adjacent non-tumour tissues. Representatives of Tspan5 staining intensity: (i) Strong in adjacent tissue, (ii) moderate in tumour tissue, (iii) weak in tumour tissue, and (iv) negative in tumour tissue. 200× magnifications, scale bar 40μm. **B.** The expression of Tspan5 was associated with GC staging. (i) Strong in adjacent normal tissue, (ii) moderate in TNM stage II, (iii) weak in TNM stage III, and (iv) negative in TNM stage IV. Upper panels: 200× magnifications, scale bar 40μm.; lower panels: 400× magnifications, scale bar 20μm. **C.** The expression of Tspan5 in 114 pairs of GC was significantly higher in tumour tissues than that of adjacent normal tissues (4.51±2.61 versus 9.66±2.30, Student's t-test, ***P<0.001). **D.** The expression of Tspan5 was inversely correlated with overall survival of all 60 GC patients (***P<0.001) as revealed by Kaplan–Meier analysis. **E.** The expression of Tspan5 was inversely correlated with overall survival of 23 patients with TNM stage I+II group (***P<0.001) or that of 37 patients with TNM stage III+IV group (**P<0.01) as shown by Kaplan–Meier analysis.

We determined the protein level of Tspan5 by immunohistochemical staining (IHC) of a cohort of 114 pairs of tumour tissues and adjacent non-tumour tissues. Tspan5 was strongly expressed in adjacent tissues but weakly expressed in tumour tissues, predominantly located on membrane and in cytoplasm of the para-neoplastic cells (Figure 1AB). Quantitative analysis by scoring the staining (Figure 1C) revealed that Tspan5 was significantly downregulated in tumour tissues versus in adjacent non-tumour tissues (4.51±2.61 versus 9.66±2.30, *P*<0.001). Based on staining scores, we divided the cohort patients into low expression group (N=55) and high expression group (N=59) respectively to investigate if the expression level of Tspan5 is associated with clinicopathological features. Interestingly, decreased expression of Tspan5 was significantly associated with tumour size (*P*<0.001), tumour invasive depth (*P*<0.05), lymph node metastasis (*P*<0.05) and TNM stage (*P*<0.01) (Table 1). However, Tspan5 expression did not appear to be associated with age, gender, tumour location and differentiation. Thus, the results suggest that decreased expression of Tspan5 may be involved in the pathogenesis of GC.

**Table 1:** Association of Tspan5 expression with clinicopathological characteristics of 114 gastric cancer patients (*χ^2^-test)

Characteristics	No. of Case	Expression of Tspan5		*P* value*
		Low	High	
Age				0.610
≥61	74	37 (50%)	37 (50%)	
<61	40 37 (50%)	18 (45%)	22 (55%)	
Gender				0.671
Male	89	42 (47%)	47 (53%)	
Female	25	13 (52%)	12 (48%)	
Tumour size (cm)				0.000
≥6	52	38 (73%)	14 (27%)	
<6	62	17 (27%)	45 (73%)	
Location				0.055
Cardiac	22	7 (32%)	15 (68%)	
Corpus	44	27 (61%)	17 (39%)	
Antrum	48	21 (44%)	27 (56%)	
Invasive depth				0.046
T1+T2	21	6 (29%)	15 (71%)	
T3+T4	93	49 (53%)	44 (47%)	
Lymph node metastasis				0.030
No	36	12 (33%)	24 (67%)	
Yes	78	43 (55%)	35 (45%)	
TNM stage				0.008
I+II	52	18 (35%)	34 (65%)	
III+IV	62	37 (60%)	25 (40%)	
Differentiation				0.127
Well/moderate	28	10 (36%)	18 (64%)	
Poor	86	45 (52%)	41 (48%)	

### Correlation of Tspan5 expression with overall survival of GC patients

Kaplan-Meier survival analysis showed that low expression of Tspan5 in GC was highly correlated with overall survival of all GC patients (*P*<0.001) (Figure 1D) or stratified patients with TNM stage I+II (*P*<0.001) and with TNM stage III+IV (*P*<0.01) (Figure 1E). Univariate Cox regression analysis demonstrated that many parameters including tumor size, invasion depth, lymph metastasis, tumour differentiation and TNM stage were significantly correlated with patient overall survival (*P*<0.05); however, others including age, gender, and tumour location were not correlated with the overall survival (Table 2). Multivariate analysis revealed that Tspan5 expression was an independent prognostic factor for GC patients (HR 6.558, 95%CI 3.055–14.078, *P*<0.001). Thus, the results suggest that decreased expression of Tspan5 may increase tumour growth and progression while increased expression of Tspan5 is an independent favourable prognostic factor for GC.

**Table 2:** Univariate and multivariate analysis of potential prognostic factors in 60 gastric cancer patients (HR: hazard ratio and CI confidence interval)

Factors	No of case	Univariate analysis		Multivariate analysis	
		HR (95% CI)	*P* value	HR (95%CI)	*P* value
Age (≥61/<61)	42/18	0.886 (0.448–1.751)	0.727		
Gender (male/female)	49/11	1.061 (0.468–2.406)	0.886		
Tumour size (≥6/<6 cm)	31/29	2.024 (1.06–3.867)	0.033		0.417
Location (Cardiac/Corpus/Antrum)	10/28/22	1.121 (0.446–2.815) 1.059 (0.522–2.146)	0.809 0.874		
Invasive depth (T1+T2/T3+T4)	9/51	0.204 (0.049–0.851)	0.029		0.456
Lymph node metastasis (No/Yes)	17/43	0.426 (0.187–0.969)	0.042		0.389
TNM stage (I+II/III+IV)	23/37	0.357 (0.172–0.738)	0.005		0.751
Differentiation (poor/well/moderate)	47/13	2.684 (1.044–6.897)	0.040		0.161
Tspan5 expression (low/high)	32/28	6.558 (3.055–14.078)	0.000	6.558 (3.055-14.078)	0.000

### Tspan5 inhibited GC proliferation, colony formation and migration *in vitro*

**Figure 2:**
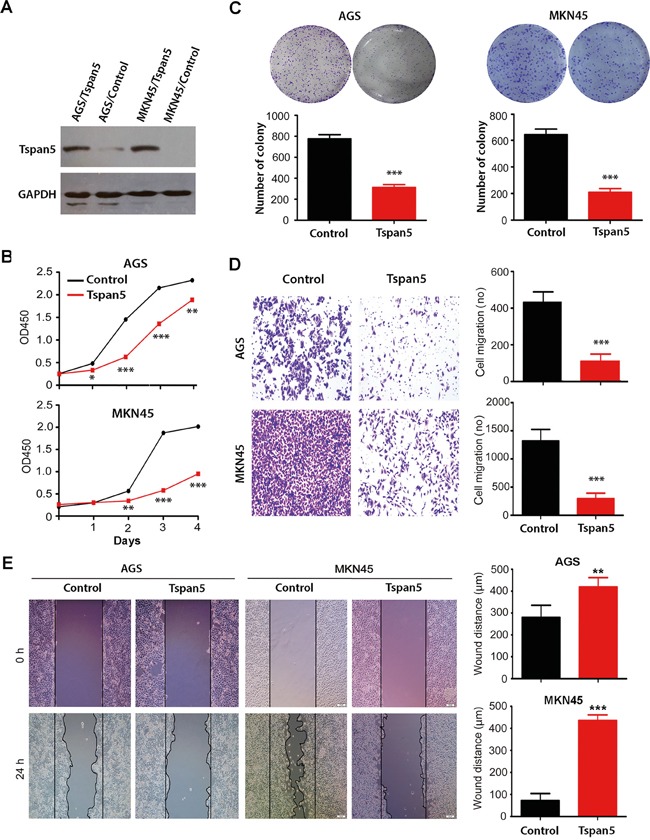
Upregulation of Tspan5 expression inhibited GC proliferation, colony formation and migration *in vitro* **A.** Western blotting confirmed the upregulation of Tspan5 in both AGS and NKN45 cell lines. AGS/Tspan5 and MKN45/Tspan5 were the AGS and MKN45 cell lines transduced with Tspan5 vector-containing retrovirus; AGS/Control, MKN45/Control were the AGS and MKN45 cell lines transduced with empty vector-containing retrovirus. **B.** CCK-8 proliferarion assays showing the proliferation inhibition of either AGS (upper panel) or MKN45 (lower panel) by Tspan5 over 4-day culture (Student's t-test, all *P<0.05). **C.** Colony formation assays showing a significant decrease of the numbers of cell colony by Tspan5 in either AGS cells (left panel) or MKN cells (right panel) (Student's t-test, ***P<0.001). **D.** Boyden chamber migration assays showing a significant decrease of GC migration by Tspan5 in either AGS cells (upper panels) or MKN cells (lower panels) (Student's t-test, ***P<0.001). **E.** Wound healing assays showing a significant decrease of GC migration by Tspan5 in either AGS cells (left) or MKN cells (right) (Student's t-test, **P<0.01).

To investigate the role of Tspan5 in pathogenesis of GC *in vitro*, we up-regulated Tspan5 expression in tumour cells by retrovirus-mediated transduction. Western blotting confirmed that Tspan5 was significantly upregulated in either AGS or MKN45 cells compared to empty vector-containing retrovirus control (Figure 2A). CCK-8 proliferation assays showed that Tspan5 inhibited cell proliferation of either AGS or MKN45 compared to relative control (Figure 2B). Colony formation assays demonstrated that Tspan5 significantly reduced the numbers of colony formed by either AGS or MKN45 cells (*P*<0.001) (Figure 2C). Boyden chamber migration assays showed that Tspan5 dramatically inhibited the migration of AGS and MKN45 toward the bottom chamber (both *P*<0.001) (Figure 2D). Consistent with the Boyden chamber results, similar results were also obtained from wound healing assays for either AGS or MKN45 cells (*P*<0.01) (Figure 2E). Thus, the results suggest that Tspan5 may act as a tumour suppressor to control tumour growth and progression of GC *in vitro*.

### Tspan5 suppressed tumor growth *in vivo*

**Figure 3:**
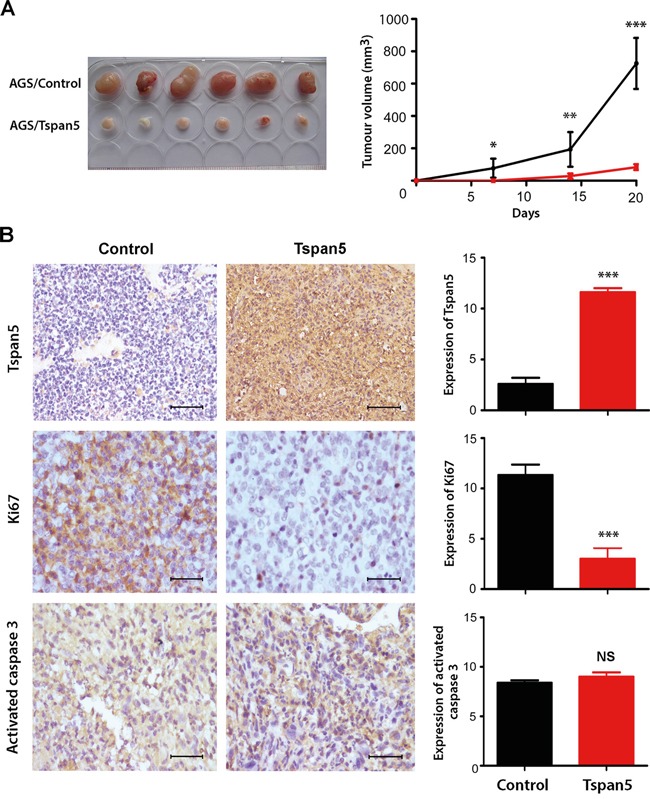
Tspan5 suppressed tumour growth of GC *in vivo* **A.** Upregulation of Tspan5 expression in AGS cells suppressed the tumour growth of GC *in vivo* (Student's t-test, *P<0.05, **P<0.01, ***P<0.001). **B.** IHC staining showing increased expression of Tspan5 but decreased expression of Ki67 in Tspan5-overexpressing tumours versus that of contro tumours (Student's t-test, ***P<0.001). However, there was no difference of the expression of active caspase 3 between Tspan5-tumour and control tumour (NS, *P>*0.05). 200× magnifications, scale bar 40μm.

To investigate whether Tspan5 affects tumour growth *in vivo*, we subcutaneously implanted Tspan5-overexpressing or control AGS cells into BALB/c nude mice [23]. Twenty days later, the animals were sacrificed and their tumours were harvested. As shown in Figure 3A, upregulation of Tspan5 significantly inhibited the tumor growth *in vivo*. Tumour volume of Tspan5-overexpressing cells was about 9-fold less than that of control cells on day 20 (84.17±7.39 versus 746.40±57.15, *P*<0.001). IHC staining of tumour sections confirmed the upregulation of Tspan5 in tumour cells compared to the control (*P*<0.001). Tumour cell proliferation was significantly decreased in the Tspan5-overexpressiing group versus in the control group (*P*<0.001), as revealed by Ki67 staining, whereas tumour cell apoptosis did not appear to have any difference between the two groups (*P*>0.05), as demonstrated by activated-caspase 3 staining (Figure 3B). Thus, the results confirm that Tspan5 functions as a tumour suppressor to inhibit tumour growth of GC *in vivo*.

### Tspan5 regulated cell cycle transition from G1-S phase by increasing p27/p15 and decreasing cyclin D1/CDK4/pRB/E2F1

**Figure 4:**
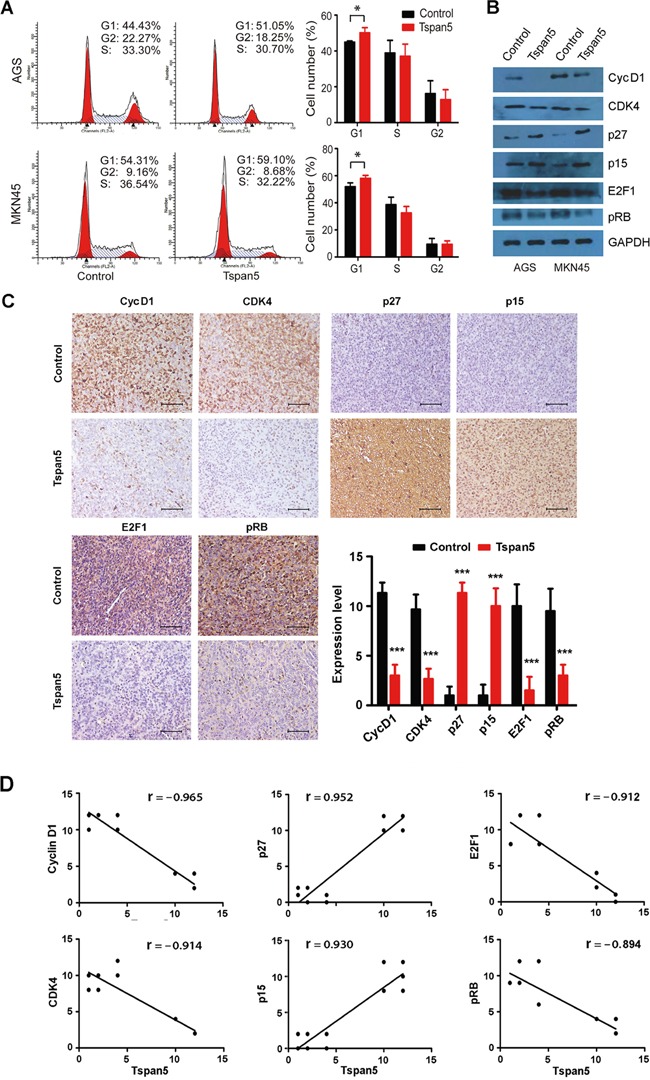
Tspan5 regulated cell cycle transition from G1-S phase by increasing p15/p27 and decreasing cyclin D1/CDK4/pRB/E2F1 **A.** Flow-cytometry analysis of the cell cycle progression showing an increase of the percentage of cells in G0/G1 phase of Tspan5-overexpressing GC compared to that of control cells in both AGS and MKN45 cell lines (Student's t-test, *P<0.05). **B.** Western blotting showing the alternation of the expression of cyclin D1, CDK4, p27, p15, E2F1 and pRB in both AGS and MKN45 cell lines by Tspan5. **C.** IHC staining showing the increased expression of p27 and p15 and the decreased expression of cyclin D1, CDK4, E2F1 and pRB in Tspan5-overexpressing tumours versus control tumours (Student's t-test, N=6, ***P<0.001). 200× magnifications, scale bar 40μm. **D.** Pearson correlation analysis for the correlation of Tspan5 expression with the expression of cyclin D1, CDK4, p27, p15, E2F1 or pRB in xenografted tumours (N=12, all ***P<0.001).

Cell cycle analysis showed that Tspan5-overexpressing AGS cells exhibited significant increased percentage of cells in G1/G0 compared to that of control cells (44.95±0.65 versus 50.14±2.89, *P*<0.05) (Figure 4A). Consistently, increased percentage of cells in G1/G0 phase by Tspan5 was also observed in MKN45 cells (51.82±2.89 versus 58.05±2.20, *P*<0.05). No significant difference for tumour cell apoptosis was observed between Tspan5-overexpressing cells and control cells in either AGS (4.61±1.22 versus 5.04±0.59, *P*=0.521) or MKN45 (8.87±0.71 versus 9.03±0.89, *P*=0.812) (Supplementary Figure). Thus, the results suggest that Tspan5 controls the cell cycle transition from G1-S phase.

We then focused on the molecular mechanisms in which Tspan5 postpones the G1-S transition by analyzing the expression of G1/S checkpoint proteins. Western blotting showed that upregulation of Tspan5 increased the expression of p27 and p15 but decreased the expression of cyclin D1, CDK4, pRB and E2F1, compared to each control, in both AGS and MNK45 cell lines (Figure 4B). To verify the *in vitro* results, we further investigated the expression of cyclin D1, CDK4, p27, p15, pRB and E2F1 *in vivo*. IHC staining demonstrated that upregulation of Tspan5 significantly increased the expression of p27 and p15 but dramatically decreased the expression of cyclin D1, CDK4, pRB and E2F1 in xenograft tumours compared to control tumours (all *P*<0.001, Figure 4C). Consistent with these results, Pearson correlation analysis revealed that the expression of Tspan5 was positively correlated with the expression of p27 (r= 0.952, *P*<0.001) and p15 (r= 0.930, *P*<0.001), but reversely correlated with the expression of cyclin D1 (r= − 0.965, *P*<0.001), CDK4 (r= − 0.914, *P*<0.001), E2F1 (r= − 0.912, *P*<0.001) and pRB (r= − 0.894, *P*<0.001) *in vivo* (Figure 4D).

### Restitution of CDK4 and cyclin D1 rescued the phenotype produced by Tspan5

**Figure 5:**
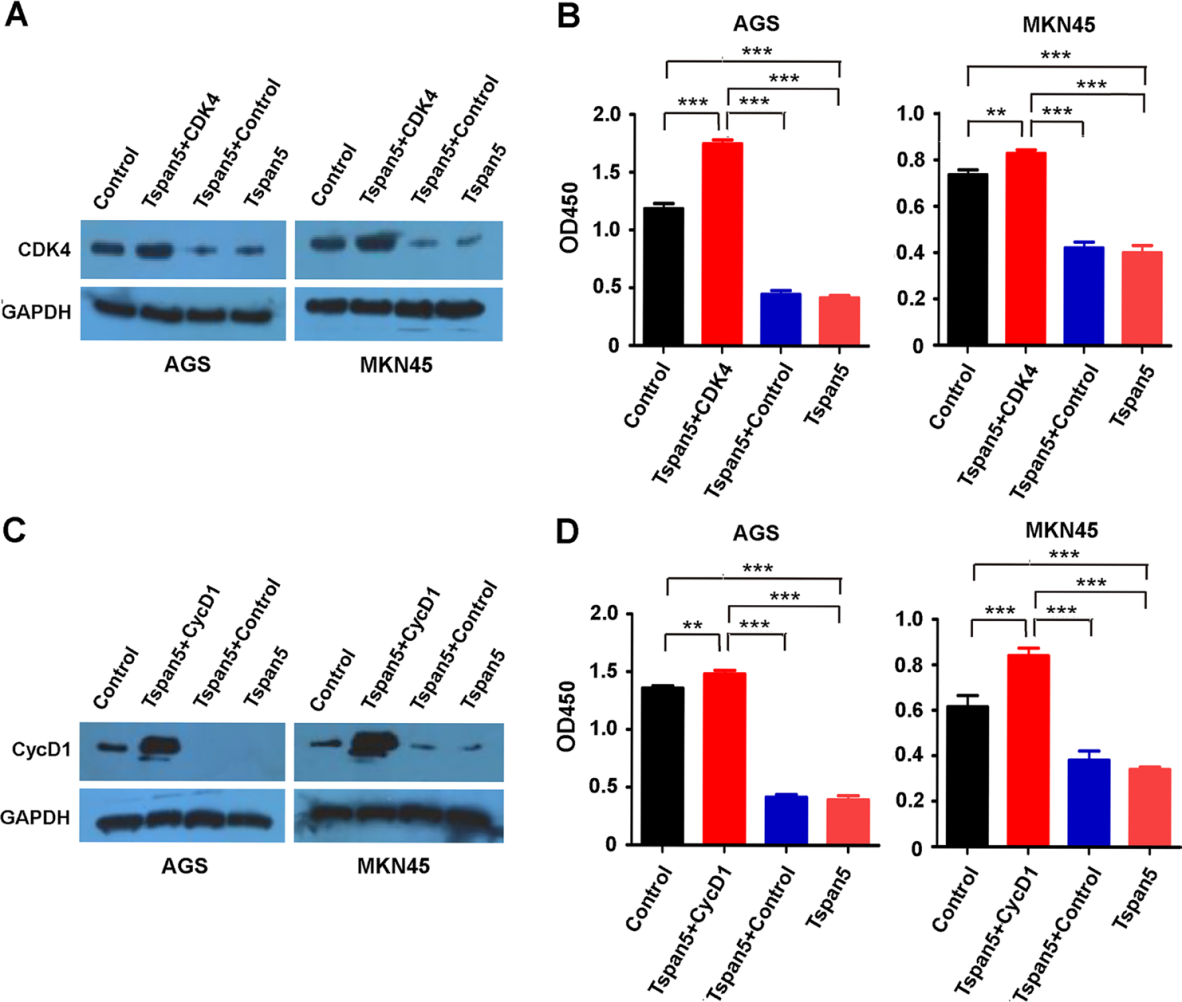
Restitution of CDK4 and cyclin D1 rescued the phenotype produced by Tspan5 **A.** Reconstitution of CDK4 in Tspan5-overexpressing (Tspan5) or control (Control) tumour cells by transfecting pCMV-Myc-CDK4 into either AGS or MKN45 cells. Western blotting confirming the downregulation of CDK4 by Tspan5 (Tspan5 versus Control) and the upregulation of CDK4 (Tspan5+CDK4 versus Tspan5+Control) in both Tspan5-overexpressing AGS and MKN45 cell lines. **B.** CCK-8 proliferation assays showing the increased proliferation of both AGS and MKN45 cells after 48 hours by reconstitution of CDK4 in Tspan5-overexpressing cells (ANOVA test, **P<0.001 or ***P<0.001). **C.** Reconstitution of cyclin D1 in Tspan5-overexpressing (Tspan5) or control (Control) tumour cells by transfecting pENTER-cyclin D1 into either AGS or MKN45 cells. Western blotting confirming the downregulation of cyclin D1 by Tspan5 (Tspan5 versus Control) and the upregulation of cyclin D1 (Tspan5+CycD1 versus Tspan5+Control) in both Tspan5-overexpressing AGS and MKN45 cell lines. **D.** CCK-8 proliferation assays showing the increased proliferation of both AGS and MKN45 cells after 48 hours by reconstitution of cyclin D1 in Tspan5-overexpressing cells (ANOVA test, **P<0.001 or ***P<0.001).

We reconstituted CDK4 in Tspan5-overexpressing tumour cells by transfecting the pCMV-Myc-CDK4 construct and confirmed the upregulation of CDK4 in tumour cells by Western blotting (Figure 5A). After 48 hours, we assessed cell proliferation *in vitro* by using CCK-8 assays. For either AGS or MNK45 cell lines, the proliferation of Tspan5+CDK4 cells was significantly increased as compared to both controls of Tspan5+Control and Tspan5 (*P*<0.001) as well as the basal control (*P*<0.01, Figure 5B). We then reconstituted cyclin D1 in Tspan5-overexpressing tumour cells by transfection with the pENTER-cyclin D1 expression vector (Figure 5C). Similarly, the proliferation of Tspan5+cyclin D1 cells was significantly increased as compared to Tspan5+Control, Tspan5 control or the basal control for both AGS and MKN4 cell lines (*P*<0.01, Figure 5D). Together, the results suggest that reconstitution of either CDK4 or cyclin D1 rescues the inhibitory phenotype produced by Tspan5, supporting further that Tspan5 suppresses the tumour growth of GC by control of the cell cycle transition from G1-S phase via decreasing the expression of CDK4 and cyclin D1.

## DISCUSSION

In this study, we demonstrated for the first time that the expression of Tspan5 is downregulated GC tumour tissues compared to adjacent non-tumour tissues. Remarkably, the expression of Tspan5 is closely associated with some clinicopathological features including tumour size, invasive depth, lymph node metastasis and TNM stage and inversely correlated with overall survival of GC patients. Multivariate analysis revealed that increased expression of Tspan5 is an independent favourable prognostic factor for predicting patient outcome in GC. Our findings suggest that decreased expression of Tspan5 may be involved in the pathogenesis of GC.

To investigate the role of Tspan5 in the pathogenesis of GC, we upregulated the expression of Tspan5 in GC cells by retrovirus-mediated transduction. We found that Tspan5 significantly inhibited the growth, colony formation and migration of GC cells *in vitro*. More importantly, upregulation of Tspan5 dramatically decreased the tumour growth *in vivo* by reducing tumour cell proliferation. Taken together, we conclude that Tspan5 functions as a tumour suppressor in stomach to control the tumour growth of GC.

Many tumour suppressors constrain cell growth and proliferation by regulating various signalling pathways that impinge the core cell cycle machinery [24, 25]. To study underlying cellular mechanisms, we performed cell cycle analysis by flow-cytometry. We found that upregulation of Tspan5 significantly increase the percentage of cells in G1/G0 phase but not in S phase and G2/M phase, suggesting that Tspan5 regulates the cell cycle transition from G1-S phase of GC. Deregulation of signalling networks during G1 phase of the cell cycle represents a major driving force in tumourigenesis and development of cancer [25]. Cyclin D1 is the first cyclin expressed in G1 phase in response to numerous mitogenic signals and peaks in mid-G1. Cyclin D1 interacts with CDK4 to form active cyclin D1/CDK4 complex that translocate to the nucleus and phosphorylate the tumour suppressor retinoblastoma (pRB). This phosphorylation leads to the release of pRB from its binding partners E2F family proteins and thus activates the transcription of E2F-dependent genes that are required for the G1-S phase transition and for the initiation of DNA replication at the late G1 [25, 26]. One mechanism that regulates the cell cycle transition from G1-S phase relies on CDK inhibitory proteins (CDKIs) such as p27^Kip^ and p15^Ink4b^ that latch onto the cyclin D1/CDK4 complex and inhibit the kinase activity [25, 27]. Therefore, CDKI, cyclin D1, CDK4, pRB and E2F encompass the central players in the cell cycle transition from G1-S phase [25, 28, 29]. To explore molecular mechanisms underlying the effect of Tspan5 on the cell cycle progression, we focused on the CDKI/cyclin D1/CDK4/pRB/E2F1 pathway. We found that upregulation of Tspan5 significantly increases the expression of p27 and p15 but decreases the expression of cyclin D1, CDK4, pRB and E2F1 in both AGS and MNK45 cell lines. More importantly, either upregulation of p15 and p27 or downregulation of cyclin D1, CDK4, pRB and E2F1 in tumour cells were also found in xenografted tumours of GC. Such upregulation or downregulation was highly correlated with the upregulation of Tspan5 *in vivo*. Indeed, upregulation of cyclin D1 and CDK4 has been found in clinical samples of GC [30, 31]. To corroborate the role of cyclin D1 and CDK4 in the suppression of tumour growth of GC by Tspan5, we reconstituted the expression of cyclin D1 and CDK4 in Tspan5-overexpressing tumour cells. We found that either cyclin D1 or CDK4 could not only reverse the inhibitory phenotype produced by Tspan5 but also promote the cell growth further as compared to the basal control, suggesting that cyclin D1/CDK4 have a dominant role in mediating the suppression of tumour growth by Tspan5 in GC.

It has been noted that TspanC8 subgroup of tetraspanins, consisting of Tspan5, Tspan10, Tspan14, Tspan15, Tspan17 and Tspan33, all six of which contain 8 cysteine residues within their main extracellular region [32, 33], were recently found to interact with a disintegrin and metalloprotease 10 (ADAM10), regulate the maturation and trafficking of ADAM10 to the cell surface [32–34], and increase the cleavage of various ADAM10 substrates, Notch signaling and ADAM10 membrane compartmentalization in multiple cell types and species [22, 32, 33, 35, 36]. In fact, ADAM10 is able to cleave the extracellular regions of more than 40 different transmembrane targets, including Notch receptors, amyloid precursor protein APP, chemokines CX3CL1 and CXCL16, growth factor receptors EGFR and VEGFR2, and various adhesion proteins [37, 38]. There is evidence that different TspanC8 proteins can promote ADAM10 shedding of specific substrates and thus impact on its substrate selectivity in different cell types [33, 36, 39]. All such information might provide some clues to further understand how Tspan5 would regulate the cell cycle transition from G1-S phase in gastric tumour. Clearly, whether Tspan5 could interact with ADAM10, increase the cleavage of its substrates such as Notch and EGFR, and thus regulate the activity of CDKI/cyclin D1/CDK4/pRB/E2F1 pathway warrants further investigation in future.

In conclusion, we have demonstrated that Tspan5 is downregulated in tumour tissue and inversely correlated with clinicopathological features and overall survival of GC patients. Increased expression of Tspan5 serves as an independent favourable prognostic factor for predicting the outcome of GC patients. Tspan5 decreases cell proliferation and colony formation *in vitro* and suppresses the xenograft growth of GC *in vivo*. Thus, Tspan5 functions as a tumour suppressor in stomach to control the tumour growth of GC. Mechanistically, Tspan5 suppresses the tumour growth through regulating the cell cycle transition from G1-S phase by increasing the expression of p27 and p15 and decreasing the expression of cyclin D1, CDK4, pRB and E2F1. Our findings provide new insights into the pathogenesis of GC and rational for the development of clinical intervention strategies against GC.

## MATERIALS AND METHODS

### Cell lines and cell culture

Human gastric cancer cell lines AGS and MKN45 were purchased from the Typical Culture Preservation Commission Cell Bank (Chinese Academy of Sciences, Shanghai, China). All cell lines were cultured in RPMI 1640 medium (Hyclone, USA) supplemented with 10% fetal bovine serum (FBS) (Gibco, UK) and maintained at 37°C with 5% CO_2_.

### Patients and clinical tissue specimens

A cohort of 114 pairs of GC tumour tissues and matched adjacent non-tumour tissues on tissue microarray (TMA) chips containing pathological and clinical information were purchased from Shanghai Outdo Biotech Co., Ltd (Shanghai, China). Patients' consent and approval from local Ethics Committee were obtained for only research purpose in use of these human clinical materials. There are 89 males and 25 females (mean age of 65 years old, ranging from 40–87 years old). All clinical samples are categorized into age, gender, tumour size, tumour location, invasive depth, lymph node metastasis, TNM stage, differentiation status, and patient survival time.

### Immunohistochemistry (IHC) and scoring

IHC staining was done with the IHC kit as described previously [40]. Briefly, the TMAs were roasted for 3 h at 65°C, deparaffinized and rehydrated through dimethylbenzene and graded alcohols, and then rinsed in tap water for few seconds. Endogenous peroxidase was blocked with 3% hydrogen peroxide for 15 min at room temperature. After rinsed in 0.01 M phosphate-buffered saline solution (PBS) for (3×3 min), the slides were then exposed to the antigen retrieval system (10 mM sodium citrate, pH6.0) in a microwave oven for 20 min. To minimize non-specific staining, the TMAs were blocked in 10 % normal goat serum for 30 min and then incubated with rabbit primary antibody anti-Tspan5 (1:600; Sigma-Aldrich, St. Louis, USA) for 2 h at room temperature. After washed in PBS (3×3 min), the slides were incubated with horseradish peroxidase (HRP)-labeled anti-rabbit secondary antibody (Gene Tech, Shanghai, China) for 30 min at room temperature and then reacted with DAB working solution (1:50; GeneTech, Shanghai, China). Hematoxylin staining was performed on the TMAs for 5 min. Histopathological features of stained tissues were evaluated and scored separately by two pathologists who blinded to the pathological information and patient clinical data. The intensity of Tspan5 staining was scored as negative (0), weak (1), moderate (2) and strong (3). The extent of Tspan5 staining was defined as the percentage of positive stained cells: 1 (<10%), 2 (10–50%), 3 (51–80%), and 4 (>80%). The final expression scores for statistical analysis were the product of the score of intensity and that of extent. The expression level of Tspan5 was considered high if the final score was ≥4 or low if the final score was <4 [40].

Xenograft tumours were also sectioned (3-4μm per section). Primary antibodies used for xenograft tumour staining were against Ki67 (1:400), active caspase 3 (1:2000), p15 (1:50), p27 (1:50), cyclin D1 (1:50), CDK4 (1:800), pRB (Ser807/811) (1:100) and E2F1 (1:50). Anti-E2F1 antibody was purchased from Bioworld Technology (USA) and others purchased from Cell Signaling Technology (USA).

### Expression vector construction and lentivirus transduction

Primers were designed to amplify the coding region of *TSPAN5* gene (NM_005723): forward 5′-CCGCTCGAGGCCACCATGTCCGGGAAGCACTACAAG-3′ and reverse 5′-CGCGGATCCCTACCAGCTCGCCCTGACAGCTTCGAT-3′. The pLNCX2-Tspan5 expression vector was constructed and verified by sequencing the inserted sequence. Either pLNCX2-Tspan5 or pLNCX2 empty vector was transfected into the packaging cell line GP2-293 with Lipofectamine 2000 (Invitrogen, USA) as described previously [40]. After 72 hours, 1 ml of viral supernatant was collected and, plus 4μg/ml of polybrene, added to AGS or MKN45 cells for stable transduction. After G418 selection (1mg/ml) for 14 days, drug-resistant cell pools were established and the protein level of Tspan5 was detected by Western blotting.

### Western blotting

Protein extractions and Western blotting were performed as described previously [40]. Primary antibodies include anti-Tspan5 (1:3000; Sigma-Aldrich) and those contained in Cell Cycle Regulation Samper Kit ½ [p15^Ink4b^, p27^Kip^, cyclin D1, CDK4, pRB (Ser807/811) and E2F1] (all 1:1000; Cell Signaling Technology, USA). GAPDH was used as a loading control to normalize the protein signal. Each experiment was repeated at least for three times.

### Cell proliferation assays

Cell proliferation assays were performed using the Cell Counting Kit-8 (CCK-8) (Dojindo, Kumamoto, Japan) according to the manufacturer's instruction. Briefly, a total of 5×10^3^ cells in 1ml were in triplicate seeded in 24-well plates and cultured at 37°C with 5% CO_2_ for one to 4 days. 20μl CCK-8 solution was added per well. After incubated at 37°C for 4 hours, absorbance at 450 nm was measured on a microplate reader (Bio-Rad). Each group was plated in three duplicate wells. Each experiment was repeated at least for three times.

### Cell cycle analysis

Approximately 1×10^6^ cells were trypsinized, washed twice with PBS, and fixed in ice-cold 70% ethanol for 1 hour. The samples were then centrifuged to remove the ethanol and exposed to 100μl RNaseA (keyGEN BioTECH, Nanjing, China) for 30 min at 37°C. Cellular DNA was stained with 25μg/ml propidium iodide (PI) (Sigma, USA). Cell cycle distributions were determined by flow-cytometry.

### Cell apoptosis assays

Approximately 1×10^6^ cells were harvested and stained with Annexin V-APC and PI according to the manufacturer's instructions (keyGEN BioTECH). Annexin V-APC/PI binding was analyzed by flow-cytometry using a BD FACSCalibur system. The data were analyzed using CellQuest software.

### Cell migration assays

Cell migration potential *in vitro* was measured using Boyden transwell chambers (8-μm pore, Corning star, Cambridge, USA). Cells in serum-free medium (5×10^4^ cells/200μl) were added to the upper chambers of transwell plates. Then 10% FBS-containing medium was added to the lower chambers as a chemoattractant. After incubation at 37°C for 24 hours, those cells that have migrated and stuck to the lower surface of the membrane were fixed with methanol and stained with 0.1 % crystal violet. For quantification, cells were counted under a microscope in five randomly selected fields (original magnification, 200×). All assays were repeated at least for three times.

### Wound healing assays

Wound healing assays were performed in 6-well plates (4×10^5^ cells/well) in which a scratch was made at the centre of each well using a plastic tip (100μl size). All undetached cells were washed away with serum-free medium. Three randomly selected identical locations were imaged at 0 and 24 h under microscopy (200×) for each replicate. Results were expressed as the distance between the edges of individual wounds at 24 hours in compared with that of t=0 point; each group included three wells. The experiment was repeated for 3 times.

### Colony formation assays

Cells in logarithmic growth phase were digested with 0.25% trypsin to acquire individual cells, which were suspended in RPMI 1640 medium containing 10% FBS. Cell suspensions of each group were diluted to 1000 cells per well (6-well plates) and cultured at 37°C with 5% CO_2_ for 2–3 weeks. When colonies became visible with the naked eye, the medium in culture wells was removed. Paraformaldehyde (4%) was added to fix cell colonies for 5min. After discarded the paraformaldehyde, 0.1% crystal violet dye was added for 15min. The dye was rinsed away with water and dried at room temperature. Numbers of cell colony were counted for statistical analysis.

### Tumour xenografts

BALB/c nude mice aged 5-6 weeks old were purchased from the Experimental Animal Centre of Southern Medical University and maintained under standard pathogen-free conditions. Tumour xenografts were performed as described previously [40]. 1×10^7^ GC cells were subcutaneously implanted into the left or right flanks of nude mice (6 mice per group). Tumour growth was measured with calipers from day 7 to day 20 after tumour cell implantation. Tumour volume was calculated by the formula of LxW^2^xπ/6, where L stands for tumour length and W for tumour width [41]. All experimental procedures were performed according to the regulation of animal usage for scientific research of Southern Medical University.

### Statistical analysis

All statistical analyses were performed using the SPSS 20.0 software (IBM Corp., Armonk, NY). Student's t-test, analysis of variance (ANOVA) or χ^2^-test were used as indicated in the results. All tests are two-sided. Kaplan–Meier analysis was used for analysis of overall survival data and Cox regression analysis for independent correlation of individual parameter with patient's overall survival. Statistical significance was indicated in the results section by an asterisk where *P*<0.05, two asterisks where *P*<0.01, or three asterisks where *P*<0.001.

## SUPPLEMENTARY FIGURE


