# Supplementary material for: Tspan5 is an independent favourable prognostic factor and suppresses tumour growth in gastric cancer
Source: Oncotarget. 2016 May 20;7(26):40160–73. doi: 10.18632/oncotarget.9514 (PMC5130000; doi:10.18632/oncotarget.9514)
Supplement: Supplementary file 1 [file oncotarget-07-40160-s001.pdf]

Tspan5 is an independent favourable prognostic factor and suppresses tumour growth in gastric cancer

SUPPLEMENTARY FIGURE

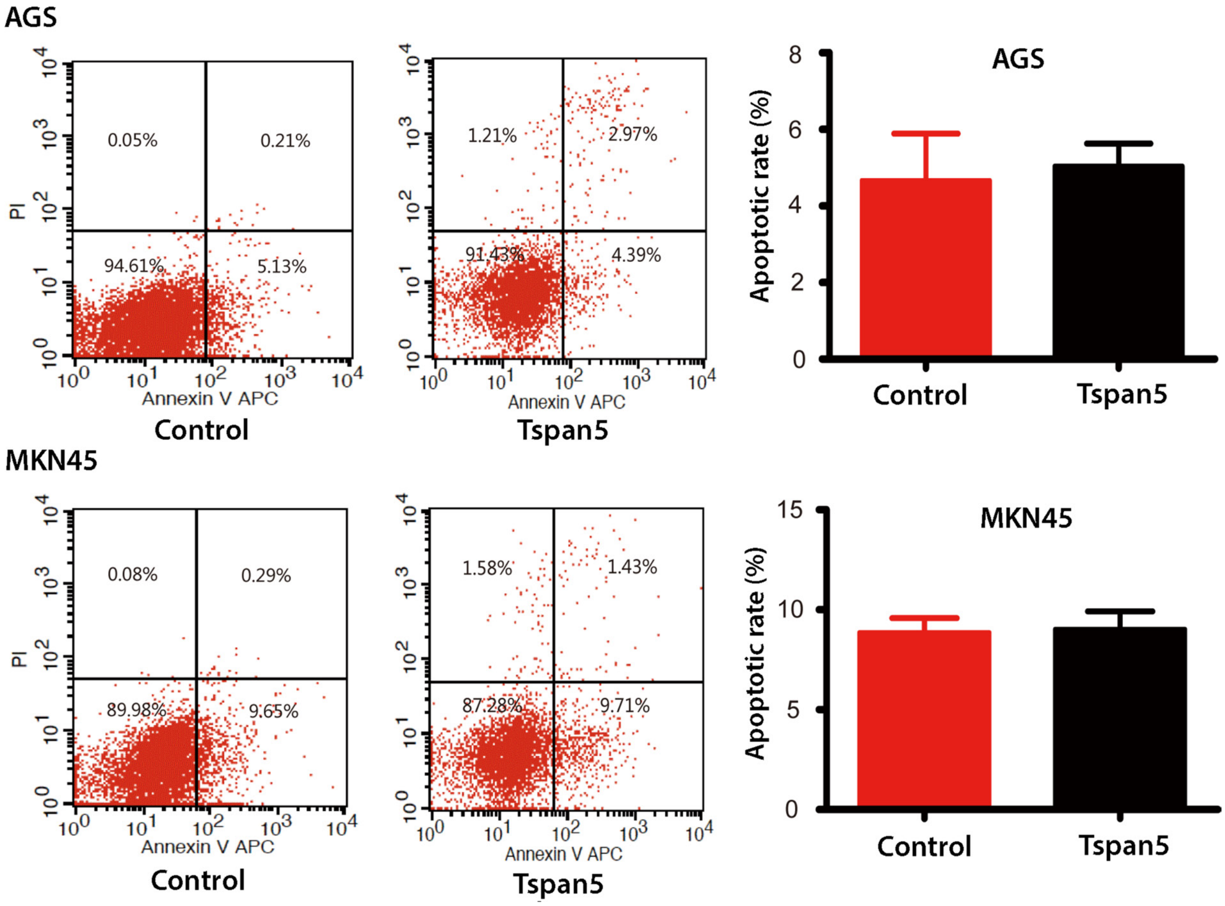

Supplementary Figure S1: Tspan5 did not affect the apoptosis of GC cells. Annexin V-APC and PI labeling apoptosis assays showed no significant difference for the percentage of apoptotic cells between Tspan5-overexpressig GC cells and the control cells of either AGS (upper panels: Student's t-test, P=0.521) or MKN45 (lower panels: Student's t-test, P=0.812).
